# Molecular profiling of cell-free DNA from classic Hodgkin lymphoma patients identifies potential prognostic clusters and corresponds with disease dynamics

**DOI:** 10.1007/s00277-025-06328-8

**Published:** 2025-04-08

**Authors:** Nick Veltmaat, Geok-Wee Tan, Yujie Zhong, Sophie Teesink, Martijn Terpstra, Johanna Bult, Marcel Nijland, Joost Kluiver, Arjan Diepstra, Anke van den Berg, Wouter J. Plattel

**Affiliations:** 1 Department of Hematology, University of Groningen, University Medical Center Groningen, Groningen, The Netherlands; 2 Department of Pathology and Medical Biology, University of Groningen, University Medical Center Groningen, Groningen, The Netherlands

**Keywords:** Hodgkin lymphoma, Liquid biopsy, Cell-free DNA, Circulating tumor DNA, Minimal residual disease, TARC

## Abstract

Cell-free DNA (cfDNA) analysis has advantages over tissue analysis for molecular profiling of classic Hodgkin lymphoma (cHL) at diagnosis and offers additional opportunities for sensitive non-invasive disease tracking during treatment. The aim of this study is to correlate cfDNA based molecular profiling with disease characteristics including serum Thymus and Activation Regulated Chemokine (TARC) levels and FDG-PET imaging, which are established markers of disease assessment. cfDNA isolated from plasma samples of 42 cHL patients was analyzed using low coverage whole genome and targeted next-generation sequencing. Patients were clustered in three groups based on Epstein-Barr virus (EBV) and *SOCS1* mutational status. Patients in the EBV-negative (EBV−) & *SOCS1* mutated (m) cluster had more extensive disease based on significantly higher serum TARC (sTARC) levels, higher metabolic tumor volume and increased risk of treatment failure. Additionally, the median variant allele frequency and mutational load was highest in the EBV− & *SOCS1*m cluster, which was validated in two external cohorts. The estimated tumor fraction and median variant allele frequency of the single nucleotide variants correlated with sTARC levels. Disease tracking over time demonstrated cfDNA level dynamics that partly resembled sTARC levels and imaging results. In conclusion, we show that cfDNA based clustering on EBV status and *SOCS1* mutational status correlates with adverse disease characteristics and increased risk of treatment failure. CfDNA-based disease tracking has the potential to serve as a sensitive tool that can complement existing response assessment methods in cHL patients.

**Supplementary Information:**

The online version contains supplementary material available at 10.1007/s00277-025-06328-8.

## Introduction

Classic Hodgkin lymphoma (cHL) is a lymphoid malignancy characterized by a paucity of multi-nucleated Hodgkin Reed-Sternberg (HRS) cells within a tumor microenvironment of inflammatory cells [1–5]. Tissue biopsies are the golden standard for diagnosis of cHL but are invasive and may not capture the full heterogeneity of the disease. Molecular profiling of cHL tumor tissue is especially challenging due to the low amount of tumor cells [6–10]. PET-CT scan analyses are commonly used for disease staging and response assessment but lack specificity and sensitivity required to detect measurable residual disease (MRD). This limits the ability to guide treatment decisions accurately [11–17]. Overall, these factors stress the need for more refined non-invasive techniques to accurately assess cHL, and guide treatment decisions effectively.

Several studies demonstrated the value of serum Thymus and Activation Related Chemokine (TARC, also known as CCL17) as a serum biomarker in cHL. Serum TARC (sTARC) levels can be elevated several years before cHL diagnosis and at diagnosis about 90% of patients show elevated levels [18, 19]. Although early response assessment using TARC in combination with FDG-PET imaging improves positive predictive value, the negative predictive value is comparable for both [20–23]. To further improve early response assessment, there is a need for a more sensitive assessment of monitoring disease activity.

Cell-free DNA (cfDNA) analysis represents a promising avenue as a novel disease biomarker in oncology. It comprises analyses of DNA fragments released into the bloodstream by both normal and tumor cells. Tumor cell derived cfDNA, which is referred to as circulating tumor DNA (ctDNA), most often originates from dead cells [24–26]. Although neoplastic cells are scarce, cHL displays elevated cfDNA and ctDNA levels [27–29]. Since it is known that ctDNA reflects the genomic landscape of HRS cells, analyzing cfDNA can offer real-time insights into the genomic landscape of a patient's tumor, providing a minimally invasive approach to monitor disease dynamics [27, 30–32]. In cHL, it has the potential to become a more sensitive method for detecting MRD than sTARC measurements [29, 33–35]. In this study, we aimed to correlate cfDNA based genomic profiling with cHL disease characteristics and dynamics as assessed by sTARC analysis and FDG-PET imaging. This may improve prognostication and response assessment and thereby guide treatment to prevent both under- and overtreatment of cHL patients.

## Methods

### Patient selection

In this study, 44 cHL patients diagnosed between 2013 until 2020 were included. Histological classification was performed according to the contemporary WHO criteria [36]. Inclusion was based on the availability of plasma samples obtained at time of diagnosis or relapse. The patient cohort was enriched for patients with relatively high metabolic tumor volume (MTV) as assessed by PET-CT scan at time of diagnosis and/or occurrence of disease relapse. sTARC levels were assessed by ELISA using routine diagnostic procedures as previously published [19]. MTV was calculated by quantifying EARL accredited PET-CT scans using 3D Slicer with the MUST-segmenter plugin with a SUV threshold of 4.0, as previously described [37].

Six of the 44 cHL patients experienced relapsed or refractory (r/r) disease and had plasma available pre-, during and after treatment. These samples were analyzed to facilitate disease monitoring. For two additional refractory cHL patients, follow-up samples were available, but they lacked pre-treatment plasma samples. Their mid- and post-treatment samples were included for the disease monitoring analysis. For these resulting eight patients, the MTV and sTARC levels were determined as a comparator for disease dynamics at the timepoints closest to plasma sampling, if available. MTV was only measured in patients with a positive FDG-PET scan defined as Deauville score 4 or 5.

Seven plasma samples from patients without malignancies were used as a control group. All patients provided informed consent for the collection and analysis of their samples including the use of clinical data. The study was conducted following the principles outlined in the Declaration of Helsinki.

### Sample processing

Blood samples were drawn into EDTA tubes and processed within a window of four hours post-collection. Plasma was isolated by centrifugation at a relative centrifugal force of 2000g for 10 min at 4 °C following established laboratory protocols. Cell-free plasma was aliquoted and conserved at −20 °C until further processing.

CfDNA was extracted from 0.38 to 2 mL of plasma using the QIAamp Circulating Nucleic Acid kit (Qiagen, Hilden, Germany). The DNA concentration was quantified using the Qubit dsDNA High Sensitivity (HS) Assay Kit, measured on a Qubit 2.0 Fluorometer (Thermo Fisher Scientific, Massachusetts, United States), and quality was assessed using Cell-free DNA ScreenTape on the 4200 TapeStation System (Agilent Technologies, California, United States).

### Immunohistochemistry

Presence of Epstein-Bar virus (EBV) in tumor cells was assessed by EBV encoded small RNA in situ hybridization (EBER-ISH) in a diagnostic setting. Additionally, available tissue samples (as indicated in the sample overview Supplementary Table S1) were stained using a polyclonal B2M (DAKO, Glostrup, Denmark) antibody in routine diagnostics. HRS cells were scored for B2M expression as previously described [38].

### Library preparation and next generation sequencing

Indexed libraries were prepared using either the Twist Library Preparation kit (Twist Bioscience, California, United States) or the SureSelect Library Preparation Kit for Illumina (Agilent Technologies), as indicated in Supplementary Table S1. Low-coverage whole genome sequencing (lcWGS) was performed on a small fraction of the library obtained before the enrichment step on a NovaSeq 6000 (Illumina, California, United States) at GenomeScan (Leiden, Netherlands), aiming for a mean coverage of 0.2x.

Target enrichment of the indexed libraries was done using a custom designed panel (244 kb) containing 72 genes commonly (hyper)mutated in B-cell lymphoma (Twist Bioscience) (Supplementary Table S2), as previously described [39]. After enrichment, libraries were amplified using 13 PCR cycles. Libraries were sequenced on NovaSeq 6000 (Illumina) at GenomeScan (Leiden, Netherlands) aiming at a mean on-target coverage of 5,000X.

### Bio-informatic analysis of next generation sequencing data and variant calling

Paired-end reads were trimmed using TrimGalore and subsequently mapped to the human genome assembly hg38 using BWA-MEM [40]. MarkDuplicates and BaseQuality Score Recalibration from the GATK toolkit were applied to identify duplicate reads and to detect systematic sequencing errors, respectively [41, 42].

Single nucleotide variants (SNVs) and small insertions/deletions (InDels) were called using an in-house pipeline, designed for calling somatic variants from cfDNA without the use of matched controls, as previously reported [39]. Pre-processed.bam files were analyzed using four variant callers (Mutect2, LoFreq, SiNVICT, VarDict). Variants were filtered on read depth (> 100), variant allele frequency (VAF) (> 1%), mutant read depth (> 7) and base quality (> 25). The remaining variants were filtered against variants observed in a Panel of Normals (PoN) of the seven control samples. SNVs overlapping with multi-nucleotide variants (MNVs) from other callers were filtered out. The resulting variants were annotated using OpenCravat and filtered based on functional effect and population frequency. Variants without a predicted functional effect, including synonymous variants and variants in 5’ or 3’ untranslated regions (UTRs), intronic regions and 2 kb up- or downstream of coding regions were excluded. Additionally, variants with a global or European allele fraction > 0.001 in the gnomAD v3 and/or 1000 Genomes databases were removed. Variants with a high VAF (> 0.20) were removed if the variant was listed with any allele frequency in the mentioned SNP databases. Additional filtering steps were applied to ensure the accuracy and biological relevance of the identified variants. InDels > 10bp were filtered to exclude those with high GC content (> 60%), or regardless of GC content for InDels > 20 bp. Variants with a base-length difference of zero and a maximum base length < 20 were retained, as these were not InDels, but rather included MNVs, comprising at least two SNVs. To address potential remaining artifacts, non-unique mutations identified in baseline samples that were not present in the COSMIC database were excluded.

For all pre-treatment samples, the median VAF of SNVs was calculated. For disease tracking over time, filtering on predicted functional effect was disregarded. The VAF cutoff applied in follow-up samples was set at > 0.004 to increase sensitivity. Variants called in the first- and at least one of the follow-up timepoints were used for tracking. ctDNA concentrations were expressed as haploid genomic equivalent per milliliter (hGE/mL) of plasma, calculated by multiplying the mean VAF for all identified re-occurring mutations by the concentration of cfDNA (pg/mL of plasma) and dividing by 3.3 (the weight of the human haploid genome, in pg), as previously described [32, 43].

Copy number variants (CNVs) were called using QDNAseq [44], with a bin size optimized for our cfDNA plasma samples of 1,000kbp. The cutoff used for calling gains/losses was set at a log 2 ratio of ± 0.08 based on the analysis of the seven control samples. The fraction of genome altered (FGA) was calculated by dividing the number of bins in CNV segments by the total number of bins. The estimated tumor fraction (ETF, fraction of ctDNA in cfDNA) was calculated using ichorCNA [45], against the merged data of the seven control samples. The maxCN setting was set to 4 to focus on low-level copy number alterations typical of cHL while minimizing noise from uncommon high-amplification states. For pre-treatment samples where the FGA was 0 based on QDNAseq data but the ETF was > 0 according to ichorCNA, the ETF value was manually adjusted to 0. For disease tracking and correlation purposes, ETF values were not manually altered.

To compare the SNV load and the median VAF between the three observed clusters (based on EBV and SOCS1 mutational status), we excluded *SOCS1* mutations. This was done to avoid potential bias, as the clusters were defined based on *SOCS1* mutational status.

### Validation of NGS findings across clusters using external datasets

To validate our clustering results, we retrieved mutational data from the studies of Maura [46] and Heger [47]. EBV status was publicly available in the Maura et al. dataset and was kindly provided by the authors of the Heger et al. dataset. We only included mutations in genes that overlapped with our sequencing panel. Patient samples were clustered in a supervised manner based on EBV and *SOCS1* mutational status.

To account for potential bias due to low ctDNA levels, SNV load was also compared between EBV− clusters after matching based on median VAF per patient. For this analysis we only included those samples that had a median VAF between the lowest median VAF in the EBV− & SOCS1m cluster and the highest median VAF in the EBV− & SOCS1wt cluster. Samples from both clusters with a median VAF falling within this range were included in the subsequent comparison of SNV load. This approach ensured that differences in SNV load were not confounded by variability in ctDNA levels across clusters.

### Statistical analysis

Baseline patient characteristics were summarized through descriptive statistics. Direct comparison between two clusters was carried out with a Wilcoxon rank-sum test. Comparison between three clusters was carried out with a Kruskal–Wallis test. Correlation between variables was assessed using Spearman correlation coefficient (ρ). A p-value < 0.05 was considered to be statistically significant. All statistical analyses were performed using open-source statistical software R (version 4.4.1).

## Results

### Patient characteristics

Our study cohort consisted of 44 cHL patients, including 19 females (43.2%) and 25 males (56.8%). The age at diagnosis ranged from 18 to 79 years, with a median of 35.5 years. Twenty-two patients presented with early-stage disease (stage I-II), and 22 had advanced-stage disease (stage III-IV). EBV status was negative in 34 patients (77.3%), positive in 9 patients (20.5%), and not determined in 1 patient (2.3%). Histological subtyping revealed mixed cellularity (MC) cHL in 6 patients (13.6%), not otherwise specified (NOS) cHL in 10 patients (22.7%), and nodular sclerosis (NS) cHL in 28 patients (63.6%). MTV ranged from 5.8 to 1,920 mL with a median of 163 mL. sTARC was elevated (> 1,000 pg/mL) in all samples and had a median of 26,000 pg/mL (range 1120 – 338,000 pg/mL). Pre-treatment plasma samples were available for 42 patients (95.5%), while follow-up plasma samples were available for 8 patients (18.2%), with six of them overlapping (Table 1).

**Table 1:** Overview of the clinical characteristics of the 44 cHL patients included in this study

	**Overall (N = 44)**
**Gender**	
Female	19 (43.2%)
Male	25 (56.8%)
**Age at diagnosis**	
Mean (SD)	38.1 (17.1)
Median [Min, Max]	35.5 [18.0, 79.0]
**Stage at diagnosis**	
I—II	22 (50.0%)
III—IV	22 (50.0%)
**EBV status**	
-	34 (77.3%)
+	9 (20.5%)
N.D	1 (2.3%)
**Subtype of cHL**	
MC	6 (13.6%)
NOS	10 (22.7%)
NS	28 (63.6%)
**MTV (mL)**	
Mean (SD)	282 (354)
Median [Min, Max]	163 [5.82, 1,920]
Missing	3 (6.8%)
**TARC (pg/mL)**	
Mean (SD)	51,500 (70,100)
Median [Min; Max]	26,000 [1,120; 338,000]
**Pre-treatment plasma available**	
No	2 (4.5%)
Yes	42 (95.5%)
**Follow-up plasma available**	
No	36 (81.8%)
Yes	8 (18.2%)

### cHL clustering based on EBV and SOCS1 mutational status

To identify the genomic profile, we analyzed cfDNA isolated from plasma of 42 pre-treatment cHL patients. An overview of total read counts, duplicate read counts and quality scores of the targeted sequencing results is presented in Supplementary Table S3. We identified variants in 38 of the 42 (91%) pre-treatment samples with a median number of seven variants (range 0–27). In these 38 patients, the median VAF per patient ranged from 1.27% to 13.11% (with an overall median of 3.4%).

**Fig. 1:**
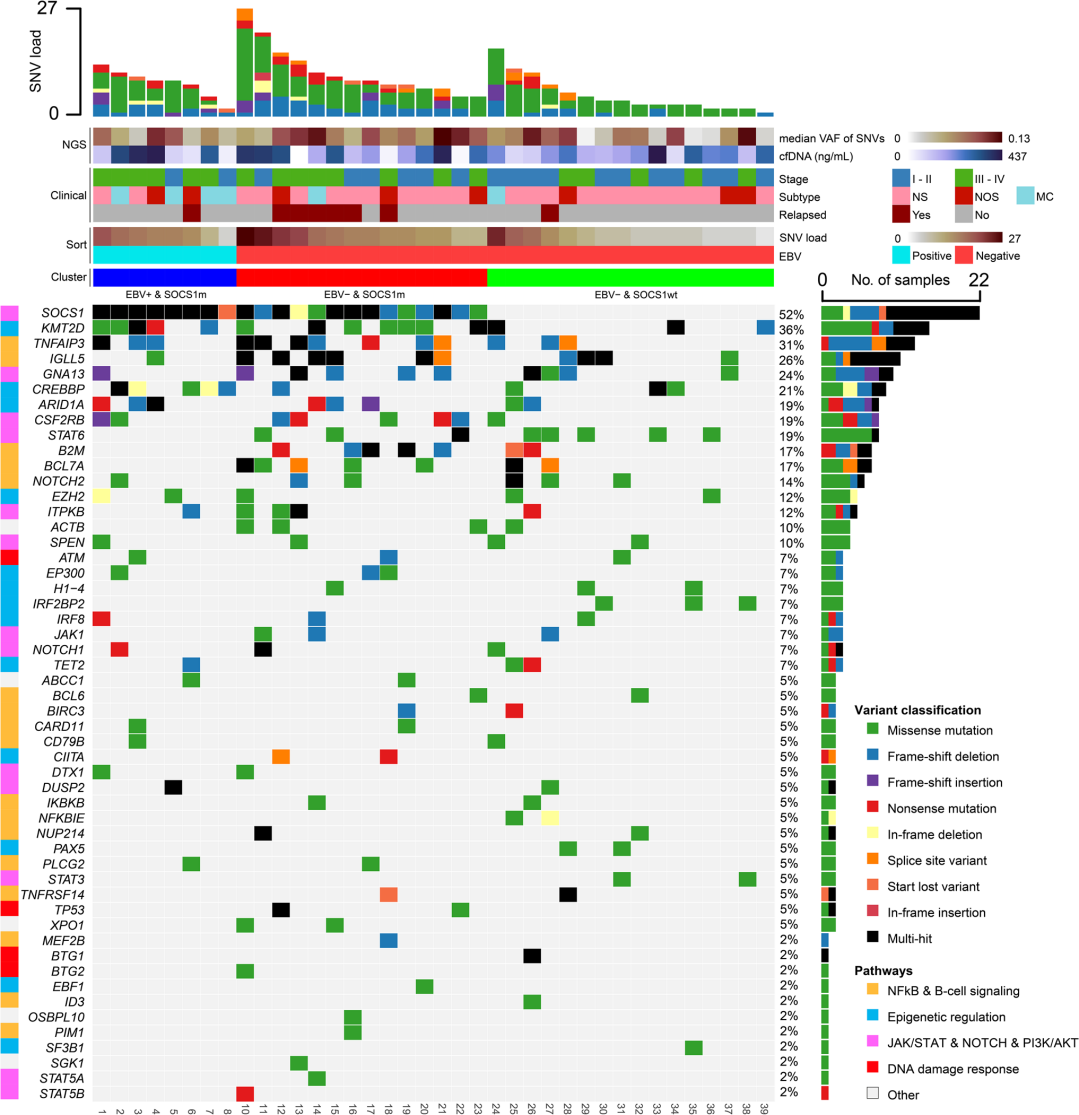
The mutational profile of single nucleotide variants (SNVs) detected in cell-free DNA (cfDNA) of classic Hodgkin lymphoma (cHL) patients. The oncoplot displays all SNVs detected per plasma sample. The top bar plot shows the total number of mutations found in each sample (SNV load) and the right-side bar shows the total number of samples with a mutation in a gene. Genes are sorted based on the number of SNVs per gene. Patients are sorted by Epstein Bar virus (EBV) status, *SOCS1* mutational status and SNV load. Three clusters were defined after sorting: EBV+ & *SOCS1* mutated (m) (blue), EBV− & *SOCS1*m (red) and EBV− & *SOCS1* wild type (wt) (green), as shown above the oncoplot. Additional information regarding whether or not a patient relapsed, cHL subtype, Ann-Arbor Stage, cfDNA concentration from plasma and median variant allele frequency (VAF) of SNVs is shown above the oncoplot. Colored boxes shown left of the gene names show the corresponding pathways. Plasma samples of patients without detectable ctDNA are not shown (n = 4)

The most frequently mutated genes in pre-treatment plasma samples were *SOCS1* in 52% of cHL patients, followed by *KMT2D* (36%), *TNFAIP3* (31%), *IGLL5* (26%), *GNA13* (24%), *CREBBP* (21%), *ARID1A, CSF2RB, STAT6* (19%), *B2M*, *BCL7A* (17%) and *NOTCH2* (14%) (Fig. 1). We observed multiple mutations for *SOCS1* in 13 out of 22 (59%) cases, and for *IGLL5* in 7 of 11 (64%) cases. The 22 *SOCS1* mutated samples harbored a total of 51 mutations, with 38 mutations in the 13 cases with multiple mutations in *SOCS1* (Supplementary Figure S1 A, B). The 11 *IGLL5* mutated samples harbored a total of 30 mutations, with 36 mutations in the 7 cases with multiple mutations in *IGLL5*. Five out of the seven samples with multiple mutations in *IGLL5* coincided with *SOCS1* mutations, of which 3 cases also harbored multiple mutations in *SOCS1* (Supplementary Figure S1 A). Mutations in the genes with multiple mutations were not localized to hotspots, but often resided within proximity to each other (Supplementary Figure S1 C, D, E). Mutations in *BCL7A* often coincided with mutations in *NOTCH2* (Supplementary Figure S1 F, Fig. 1).

We grouped the patients based on tumor cell EBV status and *SOCS1* mutational status because of its pathobiological importance. Both EBV and *SOCS1* are oncogenic drivers in cHL with *SOCS1* being the most frequently mutated gene in cHL and reported prognostic impact [48]. Strikingly, all EBV+ tumors from patients with detectable ctDNA also harbored *SOCS1* mutations, while EBV− tumors were partly *SOCS1* mutated and partly *SOCS1* wild type (wt). This resulted in three clusters: The EBV+ & *SOCS1* mutant (m); EBV− & *SOCS1*m; and EBV− & *SOCS1*wt clusters (Fig. 1). SNV load was significantly increased in the EBV− & *SOCS1*m cluster as compared to the EBV− & *SOCS1*wt cluster (p = 0.0024). The median VAF of SNVs was the highest in the EBV− & *SOCS1*m cluster (Supplementary Figure S2 A, B). To validate this clustering pattern, we applied the same clustering strategy to two previously published data sets. One dataset was derived from ctDNA sequencing [47], while the other dataset was derived from FACS-sorted HRS cells [46]. In both datasets, results similar to our findings were observed, with a significantly higher median VAF and SNV load in the EBV− & *SOCS1*m cluster (p < 0.01 [Maura et al.] & p < 0.0001 [Heger et al.]). After matching on median VAF of SNVs, the SNV load remained significantly increased compared to the EBV− & *SOCS1*wt clusters in both datasets (p = 0.037 [Maura et al.] & p < 0.001 [Heger et al.]) (Supplementary Figure S3).

### Estimated tumor fractions based on CNV correlated with VAF of SNVs

LcWGS was performed to identify CNVs in the 42 pre-treatment plasma samples. The median number of reads per sample was 3.1 million (range 0.9 – 16.8) (Supplementary Table S4). CNVs were observed in 23 out of the 42 (55%) samples. No CNVs were observed in the four patients that harbored no SNVs, likely indicating absence or extremely low levels of ctDNA. The ETF correlated significantly to the median VAF of SNVs (Supplementary Figure S4 A**)**.

**Fig. 2:**
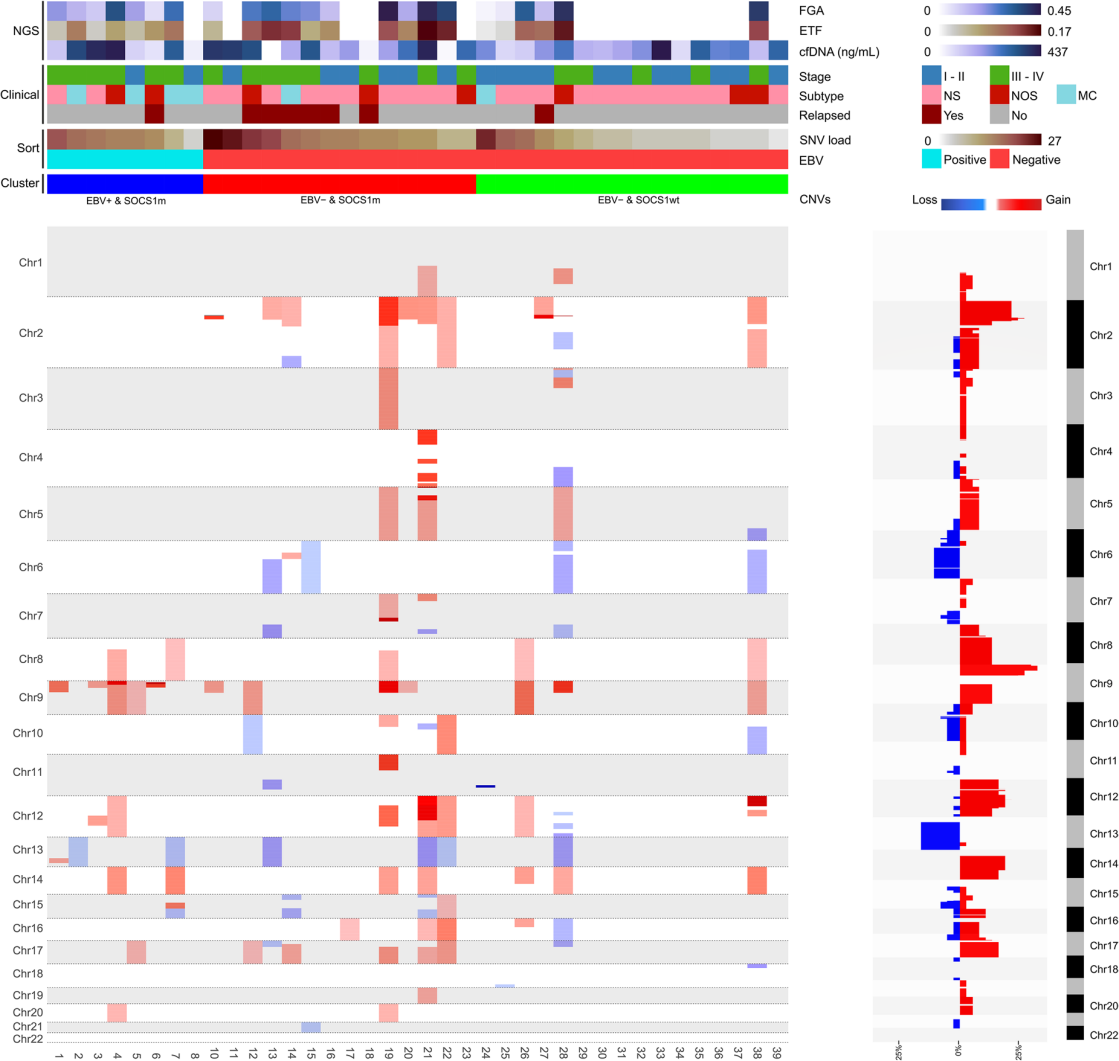
Copy number variations (CNVs) identified in cell-free DNA (cfDNA) of classic Hodgkin lymphoma (cHL) patients. Overview of CNVs for individual cHL patients, grouped by Epstein–Barr virus (EBV) status and sorted on fraction of genome altered (FGA). Chromosomal regions with gains are indicated in red and losses in blue. Frequencies of altered regions across all pre-treatment plasma samples are shown on the right. Additional information regarding cHL subtype, Ann-Arbor Stage, cfDNA concentration from plasma and estimated tumor fraction (ETF) is shown at the top of the figure. Patients without detectable ctDNA are not shown (n = 4)

The most frequently observed alterations were gains of 2p, 9p, 12, 14q and 17q, and losses of 6q and 13q. Gains at chromosome 2 and losses of parts of chromosome 6 were restricted to EBV− patients (Fig. 2). Both the ETF and FGA were significantly increased in the EBV− & *SOCS1*m cluster, as compared to the EBV− & *SOCS1*wt cluster (Supplementary Figure S2 C, D).

### Association between cfDNA NGS analyses and treatment outcome, clinical features and immune escape features in cHL

To establish the potential clinical relevance of the results of the cfDNA analyses, we compared them to sTARC levels, MTV and treatment outcome. The median VAF of SNVs correlated significantly with sTARC levels in the pre-treatment cHL samples (p = 0.0076) (Supplementary Figure S4 B) and showed a trend in the correlation with MTV (Supplementary Figure S4C). Additionally, the ETF correlated significantly with both sTARC levels and MTV (p = 0.017 & p = 0.029, respectively) (Supplementary Figure S4 D, E).

Interestingly, 6 out of 8 relapsed cases were observed in the EBV− & *SOCS1*m cluster. The remaining two relapsed cases were distributed, one in the EBV− & *SOCS1*wt cluster and one in the EBV+ & SOCS1m cluster (Fig. 1). The EBV− & *SOCS1*m cluster additionally showed significantly higher sTARC levels as compared to the other two clusters (p = 0.0029) (Supplementary Figure S2 E). This coincided with an inferior progression-free survival (PFS) of the EBV− & *SOCS1*m cluster (Supplementary Figure S5). Moreover, the MTV was also higher in the EBV− & *SOCS1*m cluster, as compared to the EBV− & *SOCS1*wt cluster (p = 0.016) (Supplementary Figure S2 F). Advanced-stage disease cases were distributed in a reasonably uniform manner across the three clusters (Fig. 1).

Next, we assessed B2M protein expression in cHL tissue sections to correlate the tumor cell expression patterns to aberrations of the genomic loci of these genes. *B2M* mutated cases were consistently negative for B2M protein expression. Conversely, no mutations in *B2M* were detected among cases with B2M protein expression (Supplementary Figure S6).

### Disease monitoring using sequential cfDNA

**Fig. 3:**
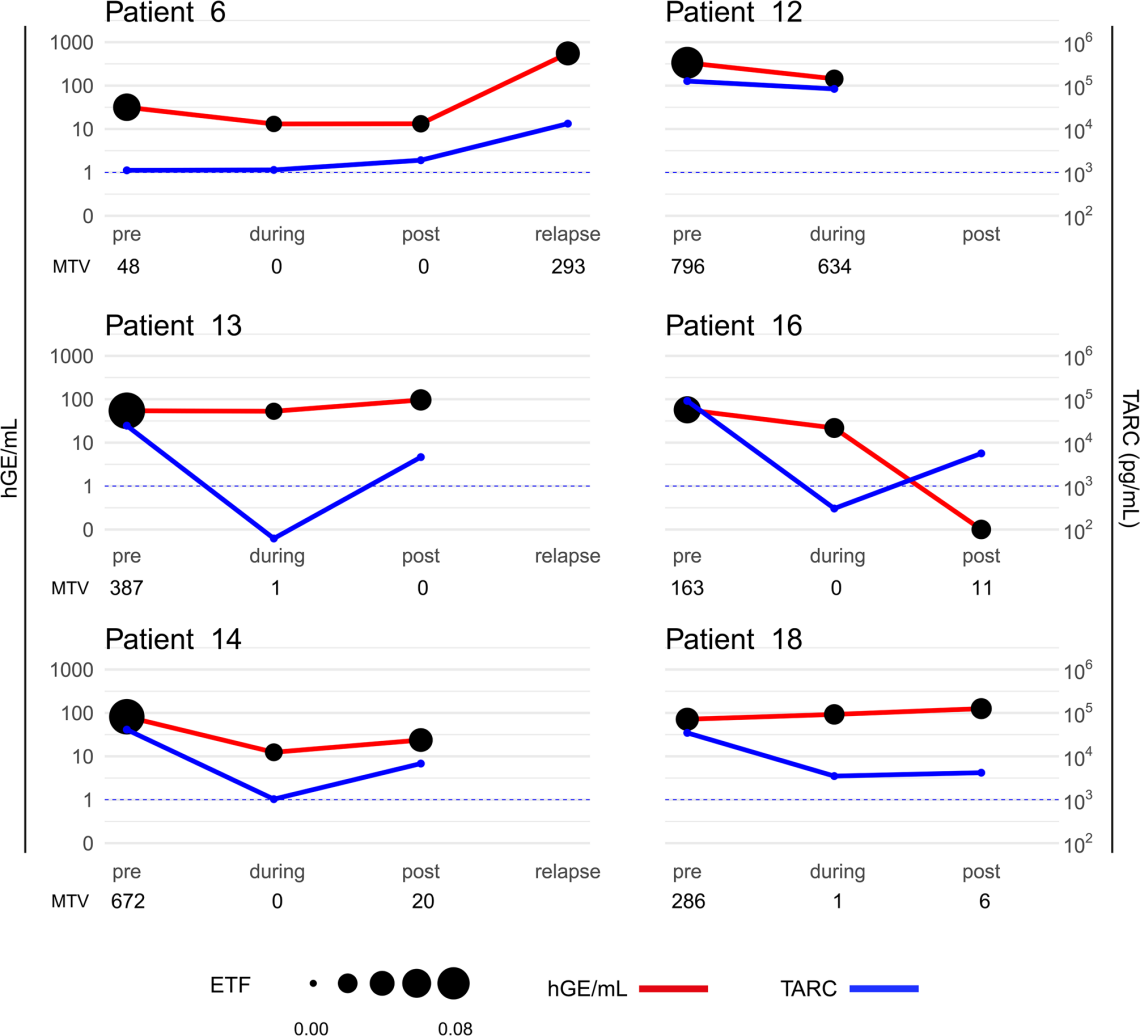
Overview of cell-free DNA (cfDNA) analysis for disease tracking over time in relation to TARC and PET. Haploid genome equivalents (hGE) of circulating tumor DNA (ctDNA) per mL plasma (red) and sTARC levels (blue) tracked over time at pre-, during and after treatment in seven patients with follow-up material. For patients 43 and 44, no ctDNA could be detected in the first available (mid-treatment) plasma sample, therefore these patients are not shown in the figure. The size of the circles shown for each hGE/mL measurement represents the corresponding estimated tumor fraction (ETF) values. Timepoints during the treatment course are indicated on the x-axes, the metabolic tumor volumes (MTV, in mL) are shown below the treatment course. Dashed blue lines indicate threshold for elevated sTARC levels (> 1000 pg/mL)

To investigate the feasibility of tracking disease dynamics using cfDNA, we analyzed sequential plasma samples from eight patients with relapsed or refractory disease. For the two patients lacking pre-treatment samples, plasma samples taken shortly after the first course of treatment were analyzed instead. Recurring SNVs and CNVs were detected in the six patients with pre-treatment samples available. In both patients lacking pre-treatment samples (# 43 & # 44), no trackable aberrations were identified in the first available plasma sample, leaving a total of six patients with trackable ctDNA (Fig. 3).

Dynamics of ctDNA levels were similar to those of sTARC and/or MTV in 5 out of 6 patients (# 6, 12, 13, 14 and 18) during and after treatment. In these patients, all having refractory or relapsed disease, a persistent detectable level of either hGE/mL or ETF in the during- and post-treatment timepoints was observed despite a negative FDG-PET at some time-points (except for patient # 12 at post-treatment, as there was no post-treatment timepoint available for this patient). Additionally, we observed a good correlation of both ETF and hGE/mL with sTARC levels in samples obtained during treatment in these five patients. Although dynamic patterns were similar, sTARC levels dropped below the threshold for elevation at mid-treatment timepoints in 2 of 6 patients (# 13 & 16) and was borderline for patient # 14, while ctDNA remained detectable. This suggests that ctDNA may have superior sensitivity as compared to sTARC in detecting MRD in these patients. ctDNA measures did not show a consistent pattern with sTARC or MTV in 1 out of 6 patients (# 16) (Fig. 3, Supplementary Figure S7).

## Discussion

In this study, ctDNA-based genomic profiling and disease tracking by ctDNA, sTARC and FDG-PET were conducted on a cohort of 44 HL patients enriched for treatment failures. We observed three clusters based on EBV and *SOCS1* mutational status. EBV is a known oncogenic driver and *SOCS1* is mutated frequently in cHL, likely playing an important role in lymphomagenesis. The EBV− & *SOCS1* mutated cluster was associated with more extensive disease, indicated by significantly higher sTARC levels and a trend for higher MTV, along with an increased risk of treatment failure. The ETF and median VAF of SNVs correlated with sTARC levels. Furthermore, for the first time, we demonstrated a correlation between ctDNA dynamics, sTARC levels, and MTV.

We and others previously demonstrated a strong overlap between both SNVs and CNVs detected in plasma and tissue samples, validating the utility of cfDNA as a marker for SNVs and CNVs even without matched normal DNA [29, 39, 49, 50]. The top mutated genes in cHL in our SNV results overlapped with previous studies describing the genomic landscape of cHL, highlighting frequent alterations in the JAK-STAT and NF-κB pathways. We did observe a higher frequency of mutations in *KMT2D* as compared to recent other genomic profiling studies in cHL. This might be explained by the high GC-content in this gene, making it more complex to accurately assess the mutational status of this gene [33–35, 46, 51–54]. The SNV load per gene was not related to gene size, as relatively small genes such as *IGLL5* and *SOCS1* had a high mutational load. This observation implies aberrant somatic hypermutation (aSHM) processes, likely involving *AID* as described by others [46, 51, 53, 55]. Additionally, we identified recurrent gains and losses in chromosomal regions frequently altered in cHL, like gains of 2p and 9p [56–58].

Our NGS analysis was clustered based on known disease drivers, i.e. EBV and *SOCS1*. The EBV− & *SOCS1*m cluster harbored cases with more adverse disease characteristics, exemplified by significantly higher sTARC levels and higher MTV levels. The association of the three identified clusters with disease extensiveness is interesting, as examining disease extensiveness using clinical staging and more recently MTV are well recognized prognostic factors in cHL [59, 60]. In line with these observations, patients with r/r disease were almost exclusively observed in the EBV− & *SOCS1*m cluster, indicating potential prognostic value of the disease driver-based clustering. At the genomic level, this cluster demonstrated a significantly higher SNV load, higher VAF of SNVs, the highest ETF and highest median FGA as compared to the two other clusters. We were able to replicate our findings with regards to a higher SNV load and median VAF in the EBV− & *SOCS1*m cluster using two publicly available cohorts [46, 47], using the same strategy as applied in our study. The cohort from Maura et al. was analyzed using FACS-sorted HRS cells, indicating that these findings are not affected by ctDNA levels, but rather by the biology of HRS tumor cells. The study of Heger et al. was analyzed using cfDNA. Furthermore, when matched for median VAF, the SNV load remained elevated in the EBV− & *SOCS1*m cluster, further implying that our findings are not confounded by ctDNA levels. The higher median VAF of the SNVs and ETF likely reflects a higher tumor burden in the EBV− & *SOCS1*m cluster. A higher SNV load and FGA might contribute to disease progression and treatment resistance [61]. These observations suggest a more aggressive disease phenotype in this cluster, potentially reflecting enhanced tumor proliferation and immune dysregulation. This was also supported by the high occurrence of *B2M* mutated cases in this cluster. *B2M* mutation status correlated with *B2M* protein expression in HRS cells, suggesting that mutations in *B2M* represent a main mechanism of immune evasion through impaired HLA-I antigen presentation, as shown previously [54, 62, 63].

The EBV− & *SOCS1*wt cluster exhibited a more favorable clinical outcome, with only one case developing a relapse. Despite the presence of genomic alterations, including SNVs, the absence of *SOCS1* mutations appears to be a prognosticator for a superior outcome of cHL patients. This finding, along with a previous study highlighting the relevance of *SOCS1* mutations for prognostication [48]*,* indicates the potential utility of *SOCS1* mutational status as a predictive biomarker for treatment outcome, especially for EBV− patients. Interestingly, all EBV+ cases harbored a mutation in *SOCS1*, except for one patient in which no ctDNA was detected. Other studies have shown that EBV+ cases tend to have less genomic alterations as compared to EBV− cases, especially in NF-κB pathways [52, 53, 64, 65]. This was only partially true in our cohort. The EBV− & *SOCS1*m cluster harbors a higher mutational load compared to the EBV+ & *SOCS1*m cluster, but EBV− & *SOCS1*wt has the lowest overall mutational load. In contrast to our findings, Alig et al. reported an unsupervised clustering method that revealed two genetically distinct clusters with superior outcome for patients with higher mutational burden, whereas the EBV− & *SOCS1*wt (low SNV load) cluster in our study has a good outcome [66].

We observed a good resemblance between ctDNA dynamics (as measured by hGE of ctDNA and ETF) and established biomarkers sTARC and MTV derived from PET-CT scans. This is the first study to directly compare these four measures, offering valuable insights. The combined analysis suggests that ctDNA dynamics can provide complementary information to the existing biomarkers. ctDNA reflects the genomic landscape of the tumor, whereas sTARC indicates disease burden and MTV measures metabolic activity. Analyzing all three offers a more comprehensive picture of disease response, potentially leading to improved clinical decision-making. Analysis of ctDNA demonstrated potential advantages in sensitivity as compared to sTARC and MTV. Identification of SNVs or CNVs during treatment in patients with low sTARC and MTV levels, underscores its potential as a more sensitive prognostic tool. This is highlighted in patients 13 and 14, where MTV and/or sTARC levels were undetectable or returned to (borderline) normal levels, respectively. Conversely, patient 16 highlights the limitations of relying solely on ctDNA, showcasing a decrease in ctDNA despite rising sTARC and MTV. These observations stress the importance of a multi-biomarker approach. By combining ctDNA with established markers like sTARC and MTV, clinicians can more accurately assess treatment response and residual disease. Future research should focus on defining the most optimal combination of biomarkers and imaging methods for sensitive and specific detection of residual disease in cHL. Additionally, advancements in ctDNA analysis methods like phased variant sequencing offer promising avenues to further enhance its sensitivity as a biomarker [67]. Integrating ctDNA analysis with other emerging technologies could provide even more detailed insights into disease dynamics and treatment response.

Despite the promising findings, there are limitations to our study. One limitation is the relatively small cohort size, which may affect the generalizability of the results. This is especially true for the r/r cases with follow-up samples, where we had sufficient material to track disease dynamics in only six patients. Although this analysis offers interesting novel insights, this part is mainly descriptive. Additionally, the relatively low frequency of cHL CNV hallmarks like 9p and 2p gains showed that the sensitivity of the lcWGS method was suboptimal. However, the relatively low costs and simplicity of lcWGS analyses may be advantageous over a targeted NGS approach. Furthermore, ctDNA levels may be too low in certain cases, particularly those with low tumor burden. A direct comparison to confirm mutational findings from ctDNA in matched tissue was not performed in this study, although previous studies have already shown good correlation between ctDNA and tissue mutational profile [29, 68]. Validation in larger, independent cohorts is necessary to confirm the prognostic value of the identified clusters and the potential utility of ctDNA dynamics as a biomarker. Larger cohorts will also allow the usage of uni- and multivariate analyses to assess prognostic value of ctDNA, sTARC and MTV for PFS and overall survival.

In conclusion, our study demonstrates that cfDNA analyses is a highly suitable, non-invasive method for molecular profiling of cHL and response monitoring. The EBV− & *SOCS1*m cluster had more extensive disease as determined by sTARC levels and MTV and showed a higher incidence of treatment failures. The correlation of ctDNA dynamics with sTARC and FDG-PET imaging during treatment underscores its potential as a treatment response biomarker. Future improvements might enhance sensitivity and increase the value of ctDNA as sensitive marker for disease activity in cHL. Ultimately, this will improve response assessment and treatment guidance in cHL patients.

## Supplementary Information

Below is the link to the electronic supplementary material.

Supplementary file1 (DOCX 683 KB)

## Data Availability

The data that support the findings of this study are available from the corresponding author upon reasonable request.
