# Supplementary material for: Molecular profiling of cell-free DNA from classic Hodgkin lymphoma patients identifies potential prognostic clusters and corresponds with disease dynamics
Source: Ann Hematol. 2025 Apr 8;104(3):1789–800. doi: 10.1007/s00277-025-06328-8 (PMC12031755; doi:10.1007/s00277-025-06328-8)
Supplement: Supplementary file 1 — Supplementary file1 (DOCX 683 KB) [file 277_2025_6328_MOESM1_ESM.docx]

# Supplementary Information

[Supplementary Information 1](#_Toc191644738)

[Supplementary Figure S1: Characteristics of the SNVs identified in pre-treatment plasma samples of cHL patients 2](#_Toc191644739)

[Supplementary Figure S2: Targeted NGS, lcWGS and clinical data compared between the three identified clusters. 3](#_Toc191644740)

[Supplementary Figure S3: Validation of EBV and cfDNA targeted NGS clusters in previously published cHL cohorts. 4](#_Toc191644741)

[Supplementary Figure S4: Correlation of cfDNA NGS results from the 42 cHL pre-treatment plasma samples vs. TARC and MTV. 4](#_Toc191644742)

[Supplementary Figure S5: Progression-free survival (PFS) compared between the three identified clusters. 5](#_Toc191644743)

[Supplementary Figure S6: *B2M* mutational status compared with protein expression. 5](#_Toc191644744)

[Supplementary Figure S7: Comparison of disease tracking efficiency of ctDNA over time to TARC and MTV. 6](#_Toc191644745)

[Supplementary Table S1: Overview of the characteristics of the 44 cHL patients. 7](#_Toc191644746)

[Supplementary Table S2: Overview of the 72 B-cell lymphoma related genes included in the targeted sequencing panel. 8](#_Toc191644747)

[Supplementary Table S3. Overview of the targeted sequencing results. 9](#_Toc191644748)

[Supplementary Table S4. Overview of the lcWGS sequencing results. 10](#_Toc191644749)


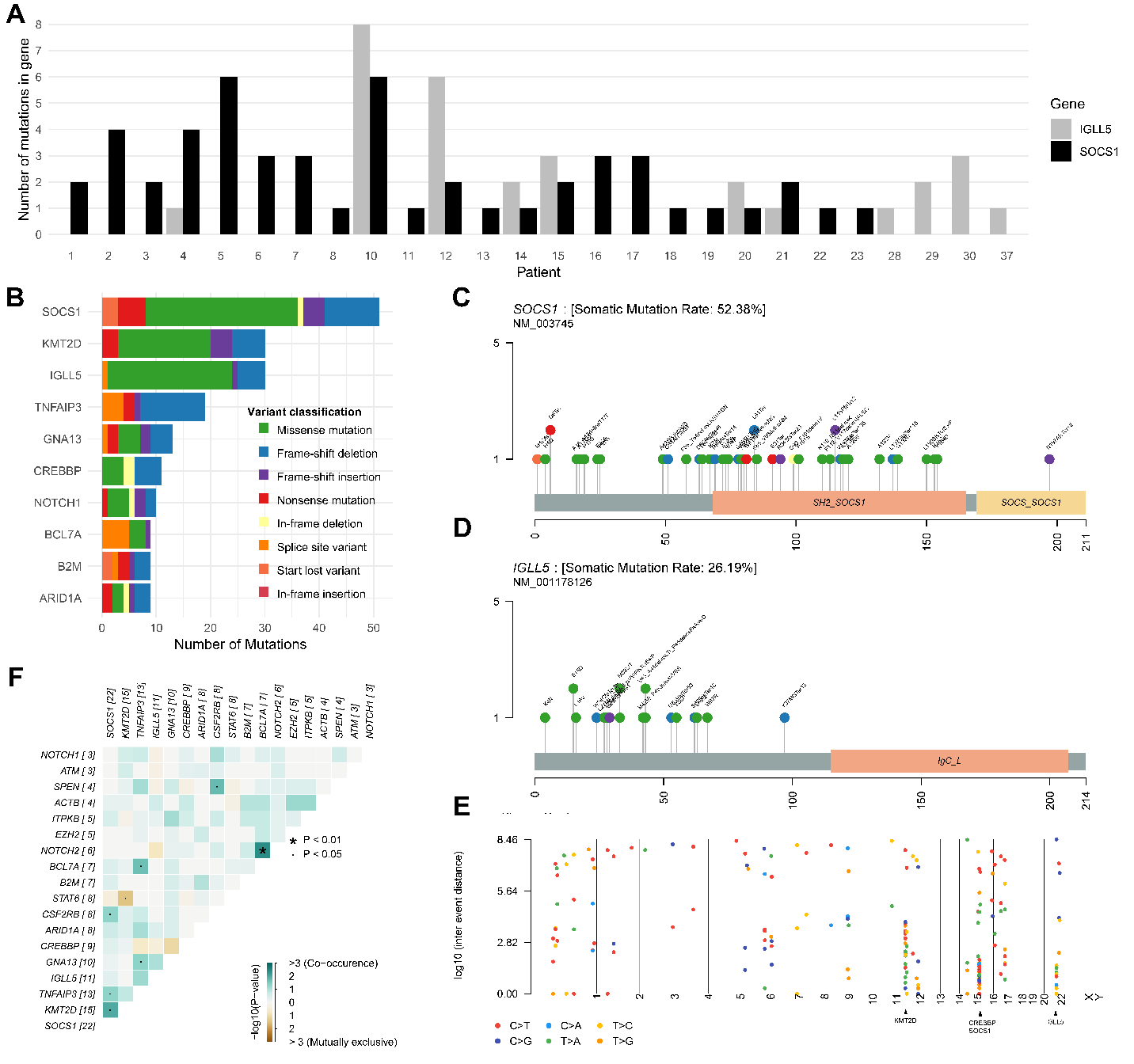


### Supplementary Figure S1: Characteristics of the SNVs identified in pre-treatment plasma samples of cHL patients

**A)** Barplot indicating the number of mutations found per sample in the frequently multi-mutated genes *SOCS1* (black) and *IGLL5* (grey). **B)** Barplot displaying the number of mutations found per gene in the top 10 mutated genes. Colors indicate the type of mutation **C, D)** Lollipop plots showing mutations across all samples in the frequently multi-mutated genes **C)** *SOCS1* and **D)** *IGLL5*. **E)** Rainfall plot showing all SNVs identified in the 42 pre-treatment plasma samples, highlighting regions where mutations lay within proximity. Dot colors indicate nucleotide substitutions. **F)** Heatmap showing co-occurrence of mutations between genes that are mutated in at least four pre-treatment plasma samples. Color indicates mutations in genes are co-mutated or mutually exclusive between samples. A dot or a star indicates significance.


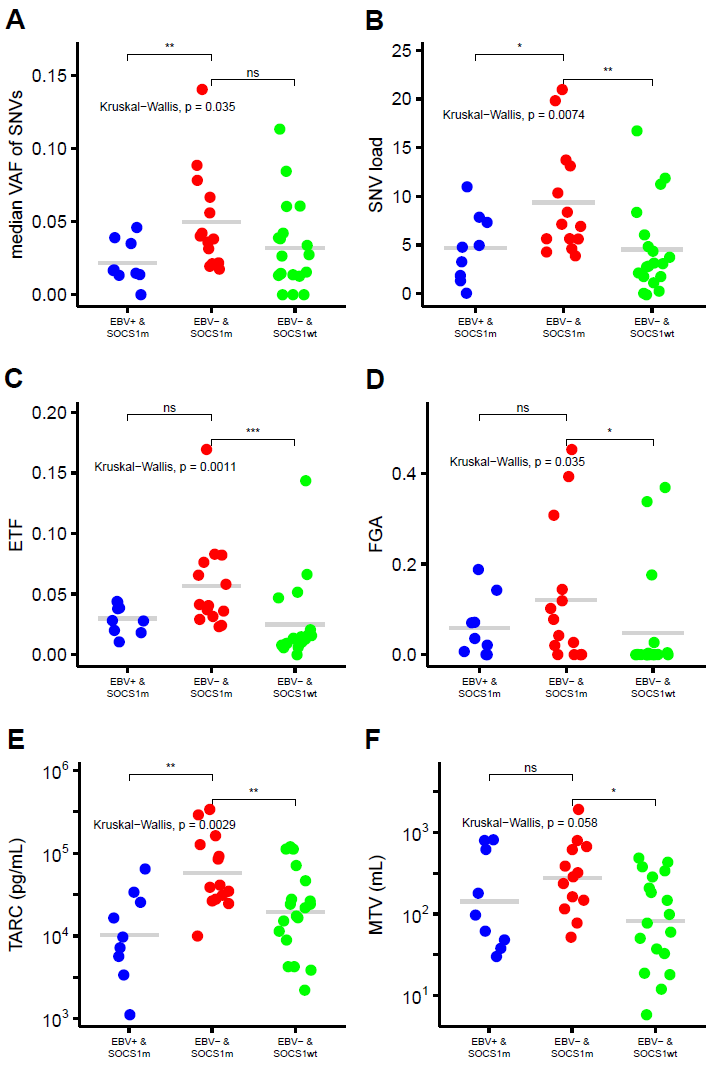


### Supplementary Figure S2: Targeted NGS, lcWGS and clinical data compared between the three identified clusters.

Comparison of **A)** SNV load, **B)** median variant allele frequency (VAF) of identified SNVs, **C)** estimated tumor fraction (ETF), **D)** fraction of genome altered (FGA), **E)** TARC level and **F)** metabolic tumor volume (MTV) between the EBV+ & *SOCS1* mutated (m), EBV− & *SOCS1*m, and EBV− & *SOCS1* wild type (wt) clusters. *SOCS1* mutations were excluded for excluded for the analysis presented in panels **A** and **B**. *, ** and *** represent p-values of ≤ 0.05, ≤ 0.01 and ≤ 0.001 respectively. Ns = not significant.


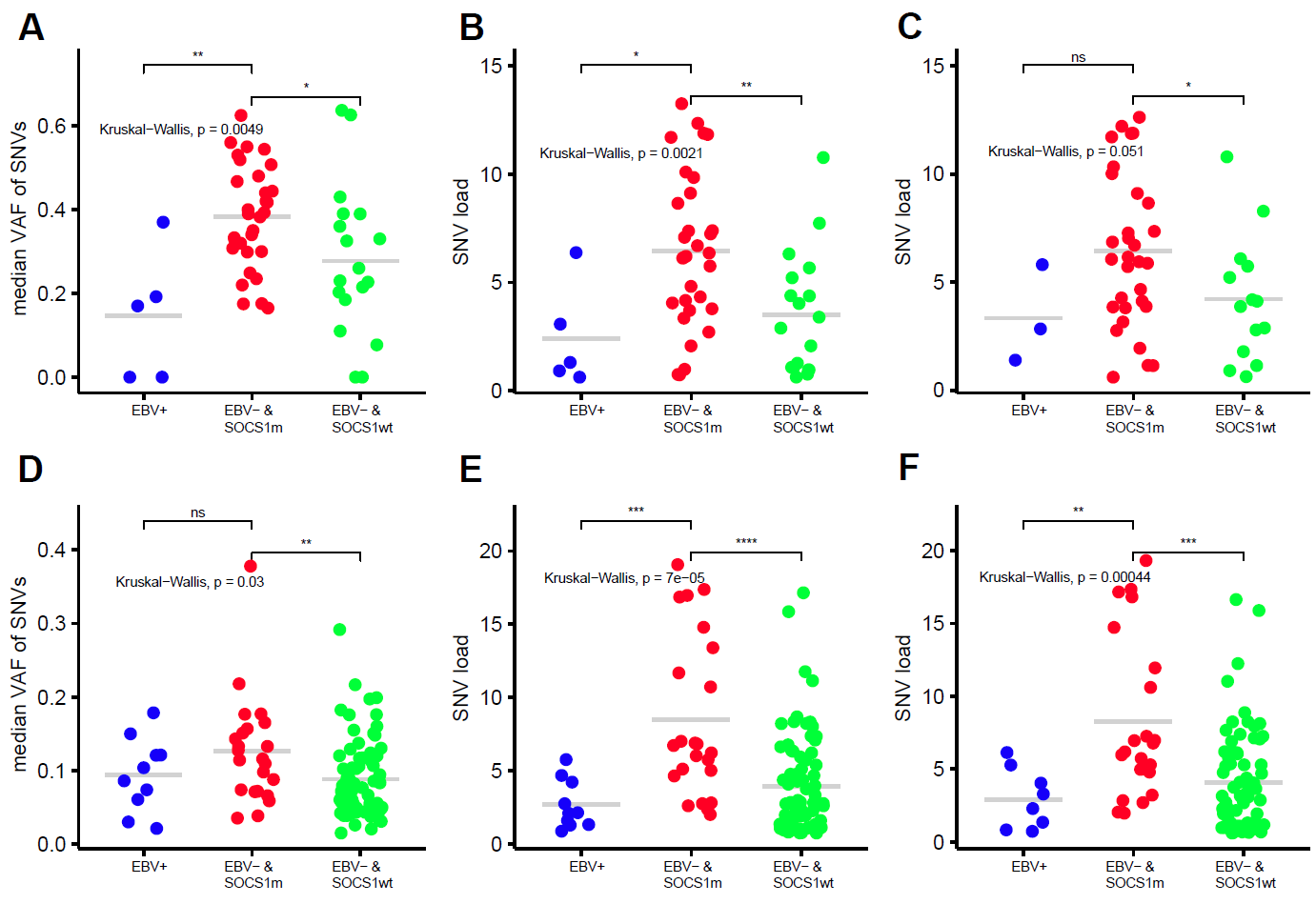


### Supplementary Figure S3: Validation of EBV and cfDNA targeted NGS clusters in previously published cHL cohorts.

Comparison of **A and D)** median variant allele frequency (VAF) of SNVs, **B and E)** SNV load and **C and F)** SNV load after matching on median VAF of SNVs between the three clusters. **A, B & C** show results of the Maura et al. cohort. **D, E & F** show the same respective results of the Heger et al. cohort.  *SOCS1* mutations were excluded for this analysis. *, **, *** and **** represent p-values of ≤ 0.05, ≤ 0.01, ≤ 0.001 and ≤ 0.0001 respectively. Ns = not significant.


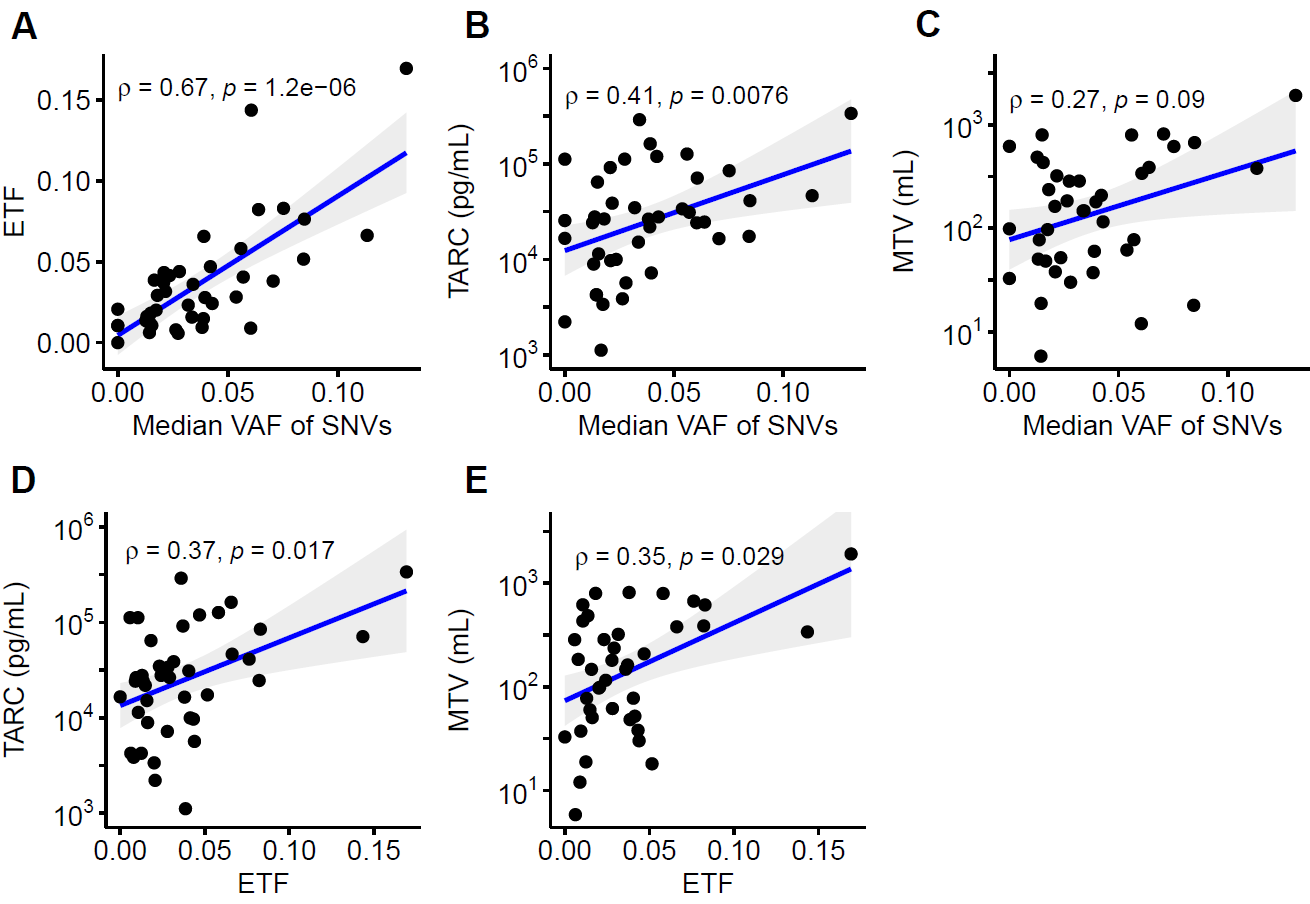


### Supplementary Figure S4: Correlation of cfDNA NGS results from the 42 cHL pre-treatment plasma samples vs. TARC and MTV.

**A)** Correlation between the estimated tumor fraction (ETF), derived from copy number variations (CNVs), and the median variant allele frequencies (VAF) of the identified single nucleotide variants (SNVs). **B, C)** Correlation of **B)** TARC and **C)** metabolic tumor volume (MTV) with the median VAF of SNVs. **D, E)** Correlation of **D)** TARC and **E)** MTV with the ETF. In all panels, the grey areas around the regression lines represent 95% CI and the Spearman coefficient is indicated with ρ.


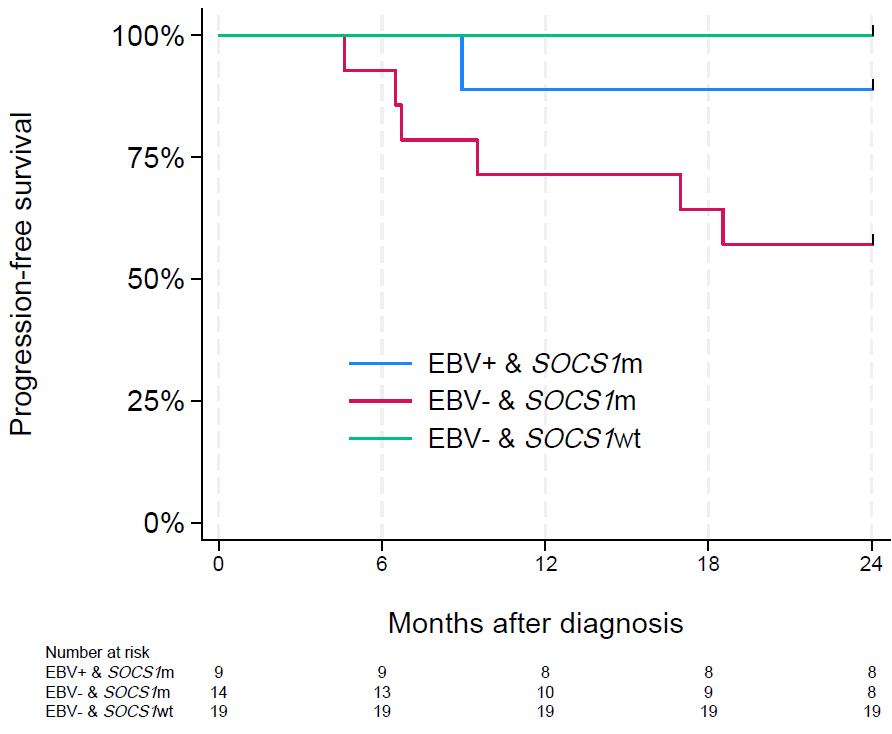


### Supplementary Figure S5: Progression-free survival (PFS) compared between the three identified clusters.

Kaplan-Meier curve displaying the differences in progression-free survival stratified for the three identified clusters as determined by EBV and *SOCS1* mutational status.


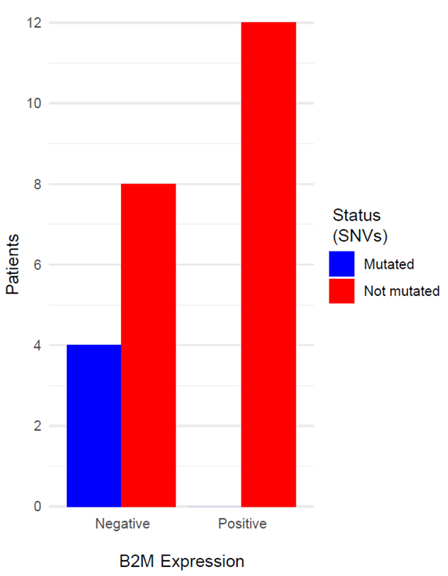


### Supplementary Figure S6: *B2M* mutational status compared with protein expression.

Bar graph displaying the relation between B2M expression and single nucleotide variants (SNVs) in the *B2M* gene. Patients with mutated B2M are shown in blue, and those with non-mutated B2M are shown in red. The x-axis represents protein expression status as determined by immunohistochemistry (IHC) and the y-axis indicates the number of patients.


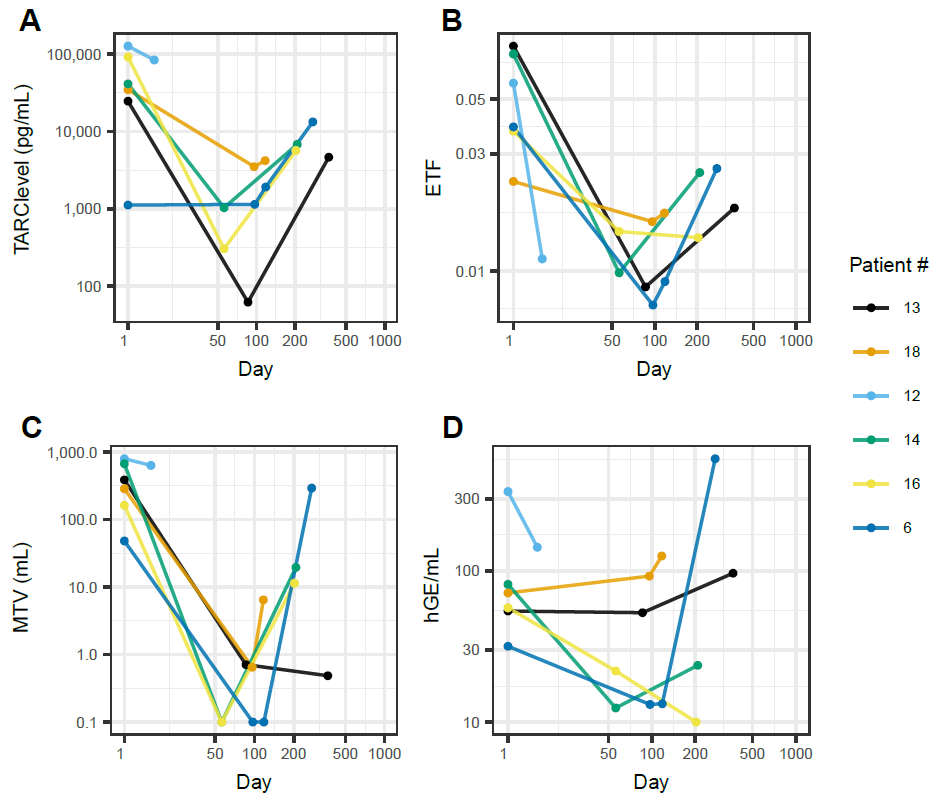


### Supplementary Figure S7: Comparison of disease tracking efficiency of ctDNA over time to TARC and MTV.

**A)** TARC levels, **B)** estimated tumor fraction (ETF), **C)** metabolic tumor volume (MTV) and **D)** human genome equivalents (hGE) of circulating tumor DNA (ctDNA) molecules per mL tracked over time for seven out of eight patients in which mutations in ctDNA were identified in the diagnostic or first plasma sample. Each color represents a patient. The overall patterns as observed appear to be mostly similar for the four plots.

### Supplementary Table S1: Overview of the characteristics of the 44 cHL patients.

| Patient No. | Pre-tx available | Time to relapse (from baseline in months) | EBER ISH | Sub-type | Stage | Age at diagnosis | Gender | TARC (pg/mL) | Library preparation | IHC available |
| --- | --- | --- | --- | --- | --- | --- | --- | --- | --- | --- |
| 1 | Yes |  | + | NS | IV | 21 | F | 7203 | Twist |  |
| 2 | Yes |  | + | MC | IV | 34 | M | 9703 | Twist | Yes |
| 3 | Yes |  | + | NS | IV | 37 | M | 64461 | Twist | Yes |
| 4 | Yes |  | + | NOS | IV | 48 | M | 16485 | Twist |  |
| 5 | Yes |  | + | MC | II | 25 | F | 33793 | Twist | Yes |
| 6 | Yes | 9 | + | NOS | IV | 62 | M | 1117 | Twist | Yes |
| 7 | Yes |  | + | MC | IV | 49 | M | 5664 | Twist | Yes |
| 8 | Yes |  | + | MC | I | 25 | M | 3378 | Twist | Yes |
| 9 | Yes |  | + | NS | IV | 50 | M | 25496 | Twist | Yes |
| 10 | Yes |  | - | NS | IV | 30 | M | 38720 | Twist | Yes |
| 11 | Yes |  | - | NS | II | 28 | M | 26472 | Twist | Yes |
| 12 | Yes | 7 | - | NOS | III | 39 | M | 126853 | Twist |  |
| 13 | Yes | 12 | - | NS | IV | 24 | F | 24614 | Twist |  |
| 14 | Yes | 7 | - | MC | IV | 65 | F | 41161 | Twist | Yes |
| 15 | Yes |  | - | NS | III | 38 | F | 290067 | SureSelect | Yes |
| 16 | Yes | 7 | - | NS | II | 20 | M | 91613 | Twist | Yes |
| 17 | Yes |  | - | NS | I | 24 | F | 31077 | Twist | Yes |
| 18 | Yes | 4 | - | NOS | IV | 55 | F | 34725 | Twist | Yes |
| 19 | Yes |  | - | NS | II | 22 | F | 162402 | Twist | Yes |
| 20 | Yes |  | - | NS | II | 27 | F | 9977 | SureSelect | Yes |
| 21 | Yes |  | - | NS | IV | 18 | M | 337579 | Twist | Yes |
| 22 | Yes |  | - | NS | II | 18 | M | 84776 | Twist | Yes |
| 23 | Yes |  | - | NOS | III | 58 | F | 27805 | Twist |  |
| 24 | Yes |  | - | MC | II | 65 | M | 3865 | Twist | Yes |
| 25 | Yes |  | - | NS | II | 40 | M | 15170 | Twist | Yes |
| 26 | Yes |  | - | NS | II | 45 | F | 17407 | Twist |  |
| 27 | Yes |  | - | NS | II | 60 | F | 119558 | Twist | Yes |
| 28 | Yes |  | - | NOS | III | 51 | M | 70960 | Twist | Yes |
| 29 | Yes |  | - | NS | III | 18 | F | 24173 | Twist |  |
| 30 | Yes |  | - | NS | II | 26 | M | 11416 | Twist | Yes |
| 31 | Yes |  | - | NS | II | 67 | M | 21894 | Twist | Yes |
| 32 | Yes |  | - | NS | IV | 23 | F | 26431 | Twist | Yes |
| 33 | Yes |  | - | NS | II | 21 | M | 4254 | Twist |  |
| 34 | Yes |  | - | NS | II | 45 | F | 24173 | Twist |  |
| 35 | Yes |  | - | NS | IV | 48 | M | 8927 | Twist |  |
| 36 | Yes |  | - | NS | II | 18 | M | 27788 | Twist |  |
| 37 | Yes |  | - | NOS | II | 21 | F | 112109 | Twist |  |
| 38 | Yes |  | - | NOS | IV | 68 | M | 46442 | Twist |  |
| 39 | Yes |  | - | NS | II | 21 | F | 4255 | Twist |  |
| 40 | Yes |  | N.D. | NS | III | 52 | M | 2215 | Twist |  |
| 41 | Yes |  | - | NS | II | 30 | M | 16585 | Twist |  |
| 42 | Yes |  | - | NOS | IV | 22 | M | 111935 | Twist | Yes |
| 43 | No | 25 | - | NOS | II | 79 | F | 22124 | Twist | N.D. |
| 44 | No | 20 | - | NS | II | 39 | F | 81326 | Twist | N.D. |

Abbreviations: tx – treatment; EBER ISH – Epstein-Barr encoding region specific RNA in situ hybridization; IHC – Immunohistochemistry; N.D. – not determined; NS – Nodular sclerosing; MC – Mixed cellularity; NOS – Not Otherwise Specified; M – Male; F – Female

### Supplementary Table S2: Overview of the 72 B-cell lymphoma related genes included in the targeted sequencing panel.

| ABCC1 | CIITA | IRF2BP2 | PLCG2 |
| --- | --- | --- | --- |
| ACTB | CREBBP | IRF8 | PRDM1 |
| AMELY | CSF2RB | ITPKB | REL |
| ARID1A | CXCR4 | JAK1 | SF3B1 |
| ATM | DDX3X | JAK2 | SGK1 |
| B2M | DTX1 | KMT2D | SOCS1 |
| BCL2 | DUSP2 | MAP3K14 | SPEN |
| BCL6 | EBF1 | MEF2B | STAT3 |
| BCL7A | EP300 | MYC | STAT5A |
| BIRC3 | EZH2 | MYD88 | STAT5B |
| BTG1 | FOXO1 | NFKBIE | STAT6 |
| BTG2 | GNA13 | NOTCH1 | TBL1XR1 |
| BTK | H1-2 | NOTCH2 | TET2 |
| CARD11 | H1-4 | NUP214 | TMSB4X |
| CD274 | ID3 | OSBPL10 | TNFAIP3 |
| CD58 | IGLL5 | PAX5 | TNFRSF14 |
| CD79A | IKBKB | PDCD1LG2 | TP53 |
| CD79B | IKZF1 | PIM1 | XPO1 |

### Supplementary Table S3. Overview of the targeted sequencing results.

| **Patient No.** | **Timepoint** | Total reads (Million) | Mapped reads (%) | Unique reads (Million) | Duplicate reads (%) | Mean Target Coverage |
| --- | --- | --- | --- | --- | --- | --- |
| **1** | **Pre** | 15 | 99,79% | 4,1 | 72,85% | 483 |
| **2** | **Pre** | 16,3 | 99,83% | 3,6 | 77,75% | 267 |
| **3** | **Pre** | 17,3 | 99,87% | 3,7 | 78,10% | 318 |
| **4** | **Pre** | 18,5 | 99,85% | 6 | 67,29% | 672 |
| **5** | **Pre** | 7,1 | 99,85% | 3,5 | 51,23% | 564 |
| **6** | **Pre** | 15 | 99,74% | 3,8 | 75,04% | 352 |
|  | **Mid** | 15 | 99,83% | 5,5 | 62,85% | 816 |
|  | **Post** | 14,2 | 99,83% | 4,6 | 67,00% | 639 |
|  | **Relapse** | 16,2 | 99,92% | 8,9 | 44,82% | 1355 |
| **7** | **Pre** | 14,9 | 99,86% | 4,2 | 71,49% | 413 |
| **8** | **Pre** | 11 | 99,84% | 3,1 | 71,66% | 416 |
| **9** | **Pre** | 9,9 | 99,89% | 5 | 49,72% | 795 |
| **10** | **Pre** | 9,5 | 99,85% | 4 | 57,25% | 667 |
| **11** | **Pre** | 15,2 | 99,85% | 3,2 | 78,61% | 239 |
| **12** | **Pre** | 9,5 | 99,90% | 4,4 | 54,10% | 670 |
|  | **Mid** | 14,8 | 99,90% | 7,4 | 49,53% | 1184 |
| **13** | **Pre** | 15 | 99,75% | 3,7 | 75,30% | 387 |
|  | **Mid** | 9,6 | 99,86% | 4,2 | 55,61% | 620 |
|  | **Post** | 9,6 | 99,88% | 2,8 | 70,92% | 325 |
| **14** | **Pre** | 10,7 | 99,85% | 5,2 | 51,40% | 846 |
|  | **Mid** | 13,2 | 99,90% | 6,7 | 48,82% | 1047 |
|  | **Post** | 11,9 | 99,50% | 3,8 | 68,13% | 302 |
| **15** | **Pre** | 9,2 | 99,59% | 5,3 | 42,70% | 685 |
| **16** | **Pre** | 7,9 | 99,84% | 3,4 | 57,28% | 545 |
|  | **Mid** | 13,8 | 99,80% | 4,4 | 67,55% | 602 |
|  | **Post** | 10,4 | 99,86% | 4,1 | 60,40% | 585 |
| **17** | **Pre** | 9,3 | 99,87% | 2,7 | 70,96% | 334 |
| **18** | **Pre** | 7,8 | 99,84% | 3 | 61,77% | 454 |
|  | **Mid** | 13 | 99,86% | 3,8 | 70,55% | 457 |
|  | **Post** | 10,4 | 99,86% | 3,1 | 69,80% | 388 |
| **19** | **Pre** | 15 | 99,76% | 4,3 | 71,43% | 444 |
| **20** | **Pre** | 11,7 | 99,30% | 3,8 | 68,14% | 260 |
| **21** | **Pre** | 7,4 | 99,85% | 4,6 | 37,12% | 839 |
| **22** | **Pre** | 15 | 99,78% | 5 | 66,78% | 600 |
| **23** | **Pre** | 8,9 | 99,83% | 2,2 | 74,70% | 290 |
| **24** | **Pre** | 15,4 | 99,79% | 3,6 | 76,28% | 308 |
| **25** | **Pre** | 15,3 | 99,89% | 6,6 | 56,89% | 949 |
| **26** | **Pre** | 7,4 | 99,86% | 1,7 | 77,39% | 180 |
| **27** | **Pre** | 8,9 | 99,90% | 3,3 | 63,56% | 451 |
| **28** | **Pre** | 13 | 99,84% | 5,9 | 54,36% | 983 |
| **29** | **Pre** | 8,8 | 99,81% | 5,4 | 38,25% | 984 |
| **30** | **Pre** | 5,5 | 99,85% | 3,2 | 42,69% | 534 |
| **31** | **Pre** | 9,2 | 99,88% | 3,8 | 58,57% | 557 |
| **32** | **Pre** | 16,1 | 99,82% | 2,7 | 82,71% | 143 |
| **33** | **Pre** | 15 | 99,82% | 4,1 | 72,98% | 454 |
| **34** | **Pre** | 6,9 | 99,89% | 1,2 | 82,47% | 108 |
| **35** | **Pre** | 7 | 99,85% | 4 | 43,42% | 722 |
| **36** | **Pre** | 11,1 | 99,78% | 4,7 | 57,18% | 791 |
| **37** | **Pre** | 4,6 | 99,90% | 1,5 | 67,22% | 192 |
| **38** | **Pre** | 13,1 | 99,88% | 7,2 | 45,44% | 1142 |
| **39** | **Pre** | 8,9 | 99,82% | 3,3 | 63,04% | 438 |
| **40** | **Pre** | 6,4 | 99,87% | 1,5 | 76,75% | 153 |
| **41** | **Pre** | 7,1 | 99,89% | 1,2 | 83,12% | 103 |
| **42** | **Pre** | 15 | 99,77% | 3,9 | 74,04% | 446 |
| **43** | **Pre** | 15 | 99,79% | 4,1 | 72,85% | 483 |
|  | **Pre** | 16,3 | 99,83% | 3,6 | 77,75% | 267 |
|  | **Pre** | 17,3 | 99,87% | 3,7 | 78,10% | 318 |
|  | **Pre** | 18,5 | 99,85% | 6 | 67,29% | 672 |
| **44** | **Pre** | 7,1 | 99,85% | 3,5 | 51,23% | 564 |
|  | **Pre** | 15 | 99,74% | 3,8 | 75,04% | 352 |
|  | **Mid** | 15 | 99,83% | 5,5 | 62,85% | 816 |
|  | **Post** | 14,2 | 99,83% | 4,6 | 67,00% | 639 |
|  | **Relapse** | 16,2 | 99,92% | 8,9 | 44,82% | 1355 |

### Supplementary Table S4. Overview of the lcWGS sequencing results.

| **Patient No.** | **Timepoint** | Total reads (Million) | Mapped reads (%) | Unique reads (Million) | Duplicate reads (%) |
| --- | --- | --- | --- | --- | --- |
| **1** | **Pre** | 2,1 | 99,01% | 1,9 | 8,81% |
| **2** | **Pre** | 16,8 | 99,74% | 15 | 10,03% |
| **3** | **Pre** | 9,1 | 99,90% | 8,5 | 6,80% |
| **4** | **Pre** | 1,7 | 99,88% | 1,6 | 8,57% |
| **5** | Pre | 2 | 99,60% | 1,8 | 10,02% |
| **6** | Pre | 3 | 99,14% | 2,6 | 9,99% |
|  | Mid | 1,9 | 99,53% | 1,7 | 9,90% |
|  | Post | 1,7 | 99,44% | 1,5 | 9,64% |
|  | Relapse | 4,6 | 99,87% | 4,1 | 8,91% |
| **7** | Pre | 3,8 | 99,80% | 3,4 | 9,80% |
| **8** | Pre | 1,4 | 99,14% | 1,2 | 9,17% |
| **9** | Pre | 4,3 | 99,71% | 3,9 | 9,89% |
| **10** | **Pre** | 2,1 | 99,84% | 1,9 | 9,21% |
| **11** | **Pre** | 1,1 | 99,77% | 1 | 9,90% |
| **12** | **Pre** | 4,4 | 99,81% | 4 | 9,90% |
|  | **Mid** | 6,4 | 99,90% | 5,7 | 10,67% |
| **13** | **Pre** | 1,5 | 99,04% | 1,3 | 9,86% |
|  | **Mid** | 5,4 | 99,59% | 4,4 | 19,58% |
|  | **Post** | 5,5 | 99,57% | 4,4 | 20,35% |
| **14** | **Pre** | 2,4 | 99,70% | 2,2 | 9,25% |
|  | **Mid** | 4,2 | 99,67% | 3,7 | 10,76% |
|  | **Post** | 1,2 | 98,42% | 1,1 | 9,58% |
| **15** | **Pre** | 4 | 99,64% | 3,6 | 10,13% |
| **16** | **Pre** | 1,7 | 99,52% | 1,5 | 9,66% |
|  | **Mid** | 1,8 | 99,24% | 1,6 | 10,09% |
|  | **Post** | 5,5 | 99,64% | 4,4 | 19,50% |
| **17** | Pre | 3,7 | 99,42% | 3,3 | 9,84% |
| **18** | Pre | 1,8 | 99,25% | 1,6 | 10,06% |
|  | Mid | 4,5 | 98,83% | 4 | 10,04% |
|  | Post | 3,7 | 99,43% | 3,3 | 10,64% |
| **19** | Pre | 3,7 | 99,45% | 3,3 | 10,18% |
| **20** | Pre | 2,4 | 98,71% | 2,1 | 8,70% |
| **21** | Pre | 3,3 | 99,87% | 3 | 9,16% |
| **22** | Pre | 1,1 | 99,47% | 1 | 8,63% |
| **23** | Pre | 2,7 | 99,34% | 2,5 | 9,25% |
| **24** | Pre | 8,2 | 99,55% | 7,4 | 9,93% |
| **25** | Pre | 6 | 99,58% | 5,3 | 10,11% |
| **26** | Pre | 3,2 | 97,74% | 2,8 | 9,87% |
| **27** | Pre | 4,2 | 99,54% | 3,8 | 10,14% |
| **28** | Pre | 4 | 99,67% | 3,6 | 9,90% |
| **29** | Pre | 3,8 | 99,78% | 3,4 | 10,00% |
| **30** | Pre | 1,5 | 99,74% | 1,4 | 8,80% |
| **31** | Pre | 4,4 | 99,63% | 3,9 | 10,30% |
| **32** | Pre | 3,5 | 99,40% | 3,1 | 10,33% |
| **33** | Pre | 1 | 99,80% | 0,9 | 8,41% |
| **34** | Pre | 3,2 | 98,21% | 2,8 | 10,36% |
| **35** | Pre | 1,9 | 99,59% | 1,7 | 8,62% |
| **36** | Pre | 2,9 | 99,55% | 2,6 | 9,66% |
| **37** | Pre | 2,1 | 99,42% | 1,9 | 9,14% |
| **38** | Pre | 4,3 | 99,76% | 3,9 | 10,14% |
| **39** | Pre | 4 | 99,52% | 3,5 | 10,52% |
| **40** | Pre | 3,1 | 98,95% | 2,8 | 10,46% |
| **41** | Pre | 3,2 | 98,45% | 2,8 | 10,63% |
| **42** | Pre | 3,3 | 99,62% | 2,9 | 10,09% |
| **43** | **Mid** | 0,9 | 98,68% | 0,8 | 10,12% |
|  | **Mid** | 1,8 | 99,54% | 1,6 | 9,45% |
|  | **Post** | 2,8 | 98,63% | 2,5 | 10,40% |
|  | **Post** | 2,1 | 99,44% | 1,9 | 9,85% |
| **44** | **Mid** | 1,6 | 96,40% | 1,4 | 10,14% |
|  | **Mid** | 5,3 | 99,58% | 4,2 | 20,10% |
|  | **Refractory** | 1,1 | 99,64% | 1,0 | 9,84% |
|  | **Post** | 1,8 | 99,68% | 1,6 | 9,66% |
|  | **Post** | 2,7 | 98,04% | 2,4 | 10,84% |
